# Tubeimoside-1 suppresses tumor angiogenesis by stimulation of proteasomal VEGFR2 and Tie2 degradation in a non-small cell lung cancer xenograft model

**DOI:** 10.18632/oncotarget.6676

**Published:** 2015-12-19

**Authors:** Yuan Gu, Christina Körbel, Claudia Scheuer, Anca Nenicu, Michael D. Menger, Matthias W. Laschke

**Affiliations:** ^1^ Institute for Clinical & Experimental Surgery, Saarland University, Homburg/Saar 66421, Germany

**Keywords:** tubeimoside-1, angiogenesis, tumor, VEGFR2, Tie2

## Abstract

Tubeimoside-1 (TBMS1) is a potent anti-tumor phytochemical. Its functional and molecular mode of action, however, remains elusive so far. Since angiogenesis is essential for tumor progression and metastasis, we herein investigated the anti-angiogenic effects of the compound. In a non-small cell lung cancer (NSCLC) xenograft model we found that treatment of CD1 nu/nu mice with TBMS1 (5mg/kg) significantly suppressed the growth and vascularization of NCI-H460 flank tumors. Moreover, TBMS1 dose-dependently reduced vascular sprouting in a rat aortic ring assay. *In vitro*, TBMS1 induced endothelial cell apoptosis without decreasing the viability of NSCLC tumor cells and inhibited the migration of endothelial cells by disturbing their actin filament organization. TBMS1 further stimulated the proteasomal degradation of vascular endothelial growth factor receptor-2 (VEGFR2) and Tie2 in endothelial cells, which down-regulated AKT/mTOR signaling. These findings indicate that TBMS1 represents a novel phytochemical for anti-angiogenic treatment of cancer and other angiogenesis-related diseases.

## INTRODUCTION

Tubeimoside-1 (TBMS1) is a major active ingredient of the Chinese medicinal herb *Bolbostemma paniculatum* (Maxim) Franquet (*Cucurbitaceae*), which is used for the treatment of snake venoms, inflammation and cancer [1]. Previous *in vitro* studies have demonstrated that TBMS1 exerts direct cytotoxicity in human cancer cell lines, such as HeLa [2], HepG2 [1, 3] and A549 [4]. In addition, TBMS1 inhibits the growth of mouse hepatoma H_22_ [5], sarcoma 180 [5, 6] and Ehrlich ascites carcinoma [5]. However, the underlying mechanisms of its anti-tumor activity have not been clarified so far.

Angiogenesis promotes tumor growth and metastasis [7]. The establishment of an adequate vascularization represents the essential step from tumor dormancy to tumor progression [8]. Excessive angiogenesis is associated with a poor prognosis for different solid tumor types, such as non-small cell lung cancer (NSCLC) [9]. During angiogenesis, endothelial cells proliferate and migrate into the surrounding tissue, which results in the formation of capillary sprouts and their interconnection to blood perfused microvessels [10]. This process is regulated by endothelial receptor tyrosine kinases, including vascular endothelial growth factor receptor (VEGFR), Tie2 (TEK), platelet-derived growth factor receptor (PDGFR) and ephrin receptor [11]. Binding of angiogenic growth factors to these receptors activates pivotal downstream signaling pathways, such as PI3K/AKT/mTOR [10, 11].

Angiogenesis is primarily driven by VEGF/VEGFR signaling [10, 12, 13]. Several agents that specifically inhibit this signaling have been approved by the Food and Drug Administration for the anti-angiogenic treatment of solid tumors [14]. Unfortunately, patients often become resistant to these agents after long-term use, which promotes the development of second-generation anti-angiogenic compounds blocking angiopoietin-2 (Ang-2)/Tie2 signaling [14]. Of interest, recent studies indicate that compounds which simultaneously inhibit both pathways are even more effective, because they exert synergistic inhibitory effects on the angiogenic process [14–16].

Based on these findings, we analyzed in the present study the anti-angiogenic effects of TBMS1. We assessed the *in vivo* action of the compound on tumor vascularization and growth in a NSCLC xenograft model. Moreover, we performed *in vitro* viability and angiogenesis assays. Finally, we investigated the intracellular mechanisms underlying the inhibitory action of TBMS1 on endothelial VEGFR and Tie2 signaling.

## RESULTS

### TBMS1 action on tumor growth and vascularization

**Figure 1:**
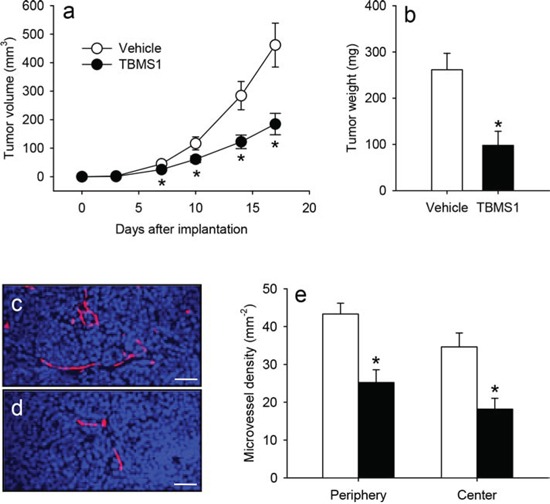
**a.** Volume (mm^3^) of developing NCI-H460 flank tumors in vehicle-treated (white circles) and TBMS1-treated CD1 nu/nu mice (black circles), as assessed by means of a digital caliper at the day of tumor induction (d0) as well as at day 3, 7, 10, 14 and 17. **b.** Final weight (mg) of the vehicle-treated (white bar) and TBMS1-treated tumors (black bar) at day 17. The data were quantified from 8 mice per group. Means ± SEM. *P<0.05 vs. vehicle. **c, d.** Immunohistochemical detection of newly formed microvessels in the center of a tumor from a vehicle-treated control mouse (c) and a TBMS1-treated animal (d) at day 17. Sections were stained with Hoechst 33342 to identify cell nuclei (blue) and an antibody against CD31 for the detection of the microvascular endothelium (red). Scale bars: 45μm. **e.** Microvessel density (mm^−2^) in the periphery and the center of vehicle-treated (white bars) and TBMS1-treated tumors (black bars) at day 17. The data were quantified from 8 mice per group. Means ± SEM. *P<0.05 vs. vehicle.

To study the anti-cancer activity of TBMS1 in a NSCLC xenograft model, human NCI-H460 cells were subcutaneously injected into the flanks of vehicle-treated and TBMS1-treated CD1 nu/nu mice. Daily administration of 5 mg/kg TBMS1 significantly reduced the volume of the developing tumors between days 7 to 17 when compared to that of vehicle-treated controls (Figure 1a). Accordingly, TBMS1-treated tumors also exhibited a markedly reduced final tumor weight (Figure 1b). Immunohistochemical analyses at day 17 revealed that the excised tumors of TBMS1-treated mice presented with a significantly lower density of CD31-positive microvessels in their periphery and center when compared to those of vehicle-treated animals (Figure 1c-1e).

These results demonstrate that TBMS1 efficiently suppresses tumor growth and vascularization in the NSCLC xenograft model. Noteworthy, the animals tolerated the daily treatment with TBMS1 well, as indicated by normal feeding, cleaning and sleeping habits, which did not differ from those of vehicle-treated controls.

### TBMS1 action on vascular sprouting

**Figure 2:**
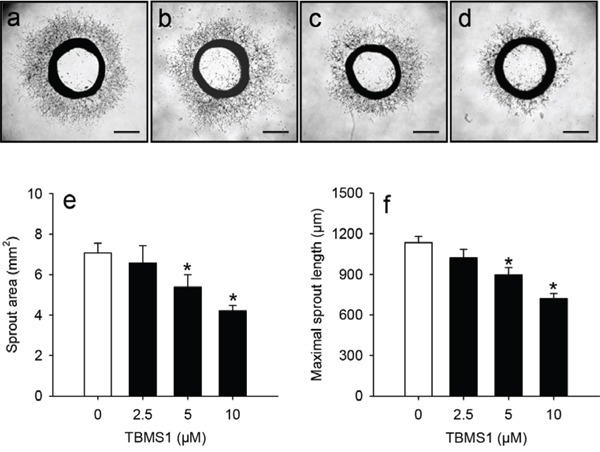
**a-d.** Phase contrast microscopic images of rat aortic rings exposed for 6 days to vehicle (0μM; a), 2.5 (b), 5 (c) and 10μM TBMS1 (d). Scale bars: 1mm. **e, f.** Sprout area (mm^2^) (e) and maximal sprout length (μm) (f) of the outer aortic vascular sprouting at day 6 after incubation of aortic rings, as assessed by computer-assisted image analysis. The rings were exposed to vehicle (0μM; white bars) and 2.5, 5 and 10μM TBMS1 (black bars). The data were quantified from 8 aortic rings per group. Means ± SEM. *P<0.05 vs. vehicle.

To prove a direct anti-angiogenic effect of TBMS1 in an experimental setting excluding the influence of tumor cells, we next performed an *ex vivo* aortic ring assay. In this assay, incubation of rat aortic rings in Matrigel stimulates the growth of vascular sprouts out of the aortic wall. We found that this angiogenic process was dose-dependently suppressed by TBMS1 (Figure 2a-2d). After 6 days incubation aortic rings exposed to TBMS1 presented with a significantly reduced sprout area and maximal sprout length when compared to vehicle-treated controls (Figure 2e and 2f).

### TBMS1 action on viability of endothelial cells and tumor cells

Both the inhibition of angiogenesis and the direct cytotoxicity of TBMS1 against tumor cells may have contributed to the observed tumor shrinkage in our NSCLC xenograft model. To further unravel the specific effects of TBMS1 on these processes, we assessed the viability of TBMS1-treated murine endothelial eEND2 cells, human dermal microvascular endothelial cells (HDMEC) and the two human NSCLC cell lines NCl-H460 and A549 by means of water-soluble tetrazolium (WST)-1 assays and flow cytometry.

**Figure 3:**
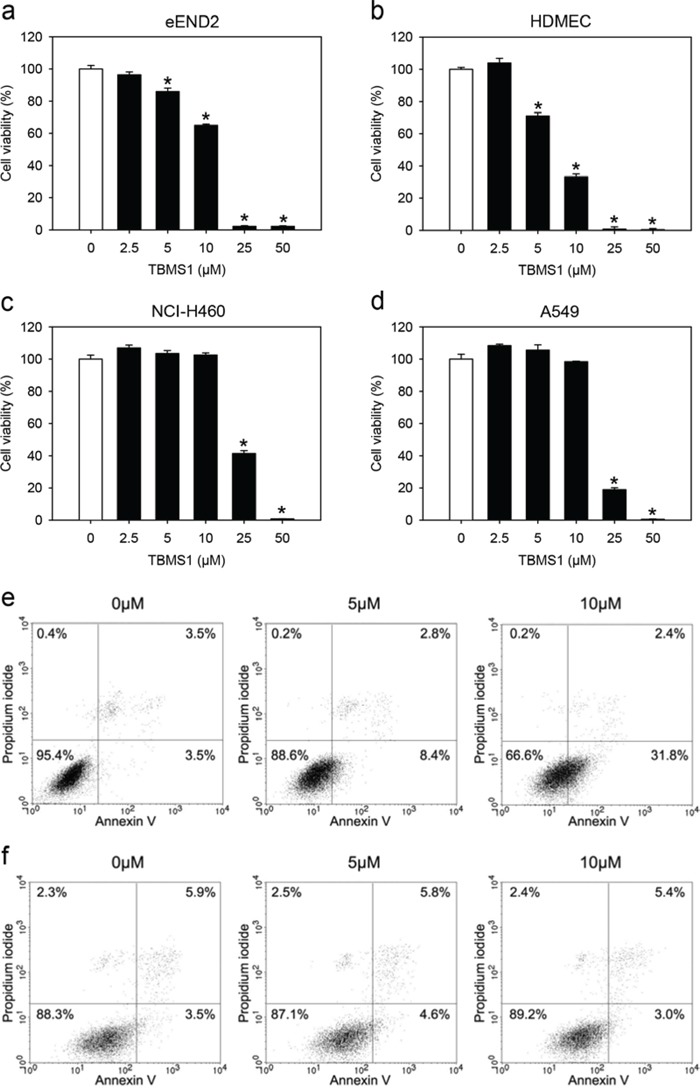
**a-d.** Cell viability (%) of eEND2 cells (a), HDMEC (b), NCI-H460 cells (c) and A549 cells (d), which were exposed for 24h to different doses (2.5 to 50μM; n=4) of TBMS1 or vehicle (0μM; n=4), as assessed by WST-1 assay. The data represent 3 independent experiments with 4 repeats. Means ± SEM. *P<0.05 vs. vehicle. **e, f.** Representative flow cytometry histograms of 3 independent experiments. eEND2 cells (e) and NCI-H460 cells (f), which were exposed for 24h to 5 and 10μM TBMS1 or vehicle (0μM), were stained with propidium iodide and annexin V.

Because in our xenograft model microvessels of murine origin invaded the developing tumors, we first analyzed the viability of murine eEND2 cells. Our results showed that doses of 5 to 50μM TBMS1 significantly reduced the viability of these cells in the WST-1 assay with a half maximal inhibitory concentration (IC50) of 12.0μM (Figure 3a). Similar results were found for HDMEC, which were used as control endothelial cells of human origin (Figure 3b). These cells reacted even more susceptible to the exposure of TBMS1 with a IC50 of 7.3μM. In contrast, the cell viability of NCl-H460 cells was only reduced when exposed to high concentrations of 25 and 50μM of TBMS1 (Figure 3c). Accordingly, the IC50 of the compound was markedly higher (23.3μM) for these tumor cells when compared to the two analyzed endothelial cell types. To exclude that this finding was only specific for NCl-H460 cells, we tested another NSCLC tumor cell line, i.e. A549. Here, we also found that the cell viability of A549 cells was only reduced when exposed to 25 and 50μM of TBMS1 with a IC50 of 18.1μM (Figure 3d).

Flow cytometric analyses revealed that doses of 5 and 10μM TBMS1 increased the number of annexin V-positive/propidium iodide-negative early apoptotic eEND2 cells (Figure 3e). However, in line with our WST-1 assays, exposure to these doses of TBMS1 did neither induce apoptotic nor necrotic cell death in NCl-H460 cells (Figure 3f). Taken together, these results indicate that endothelial cells react more sensitive to TBMS1 treatment than NSCLC tumor cells.

### TBMS1 action on migration of eEND2 cells

**Figure 4:**
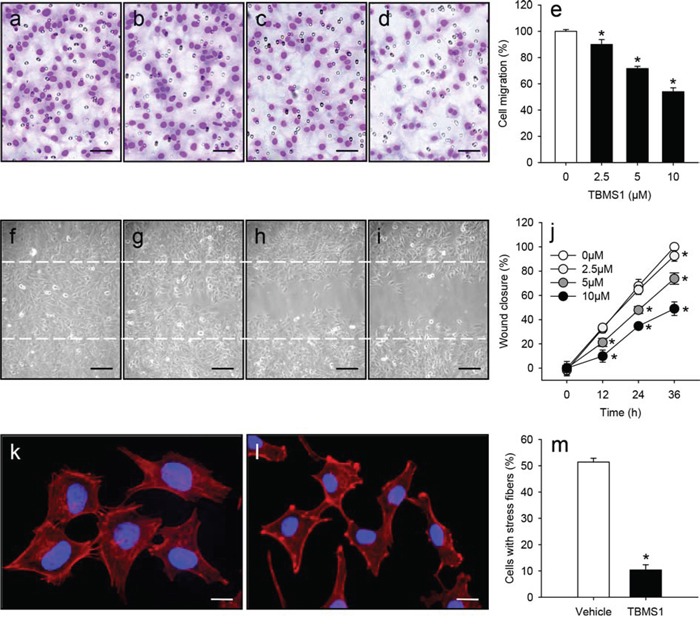
**a-d.** Light microscopic images of eEND2 cells, which have migrated and attached to the bottom membrane of the transwell migration assay. The cells were treated for 24h with vehicle (0μM; a), 2.5 (b), 5 (c) or 10μM TBMS1 (d) followed by the transwell migration assay. Scale bars: 60μm. **e.** Cell migration (%) of eEND2 cells, which were treated for 24h with vehicle (0μM; white bar) or 2.5, 5 and 10μM TBMS1 (black bars), as assessed by the transwell migration assay. The data represent 4 repeats per group. Means ± SEM. *P<0.05 vs. vehicle. **f-i.** Phase contrast microscopic images of eEND2 cells, which were scratched and subsequently treated for 36h with vehicle (0μM; f), 2.5 (g), 5 (h) or 10μM TBMS1 (i). Dashed lines indicate original wound size. Scale bars: 120μm. **j.** Wound closure (%) of scratched eEND2 cells at 0, 12, 24 and 36h, as assessed by the scratch wound healing assay. The cells were exposed to vehicle (0μM; white circles), 2.5 (light grey circles), 5 (dark grey circles) or 10μM TBMS1 (black circles). The data represent 4 repeats per group. Means ± SEM. *P<0.05 vs. vehicle. **k, l.** Fluorescence microscopic images of eEND2 cells, which were treated for 24h with vehicle (k) or 10μM TBMS1 (l). The cells were stained with Alexa Fluor 568-conjugated phalloidin (red) for the detection of the cytoskeleton. The cell nuclei were stained with Hoechst 33342 (blue). Scale bars: 17μm. **m.** Cells with stress fibers (%) in the group of vehicle-treated (white bar) and TBMS1-treated (black bar) eEND2 cells. The data represent 3 independent experiments with 3 repeats. Means ± SEM. *P<0.05 vs. vehicle.

We next investigated the effects of TBMS1 on the migratory activity of eEND2 cells by means of a transwell migration assay and a scratch wound healing assay. We found that TBMS1 reduced the number of migrated eEND2 cells in the transwell migration assay (Figure 4a-4e). Moreover, TBMS1 significantly delayed the wound closure in the scratch wound healing assay in a time- and dose-dependent manner (Figure 4f-4j).

Endothelial cell migration is driven by the continuous remolding of the actin cytoskeleton with formation of filopodia, lamellipodia and stress fibers [17]. Hence, we additionally performed phalloidin staining of both vehicle-treated and TBMS1-treated eEND2 cells (Figure 4k and 4l). We could demonstrate that TBMS1 treatment significantly decreased the number of cells with stress fibers (Figure 4m). This indicates that the compound inhibits endothelial cell migration by disturbing actin filament organization.

### TBMS1 action on VEGFR2 and Tie2 signaling

**Figure 5:**
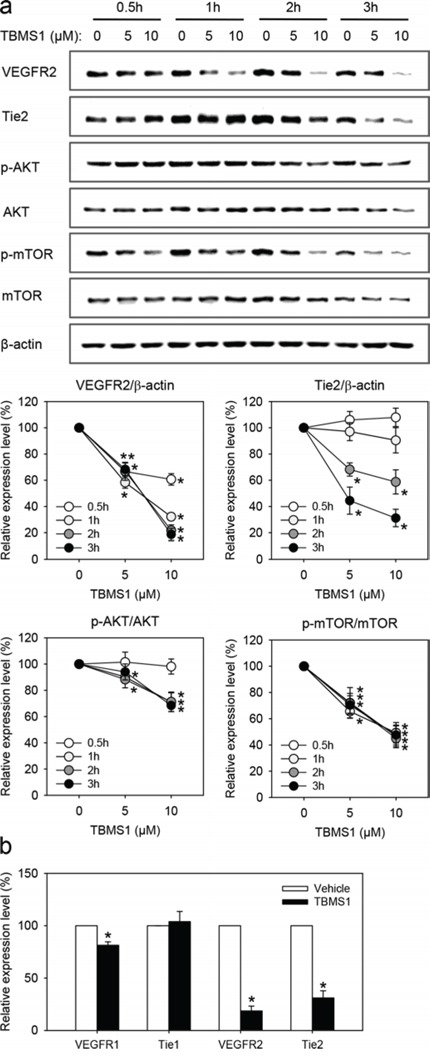
**a.** Western blot analysis of VEGFR2, Tie2, p-AKT/AKT and p-mTOR/mTOR expression of eEND2 cells (% of control at each time point), which were treated for 0.5h (white circles), 1h (light grey circles), 2h (dark grey circles) and 3h (black circles) with vehicle (0μM; control), 5 and 10μM TBMS1. The data were quantified from 3 independent experiments. Means ± SEM. *P<0.05 vs. vehicle. **b.** Western blot analysis of VEGFR1, Tie1, VEGFR2 and Tie2 expression of eEND2 cells (% of control), which were treated for 3h with vehicle (0μM; control) or 10μM TBMS1. The data were quantified from 3 independent experiments. Means ± SEM. *P<0.05 vs. vehicle.

To delineate the molecular mechanisms underlying the anti-angiogenic effect of TBMS1, we performed Western blot analyses of eEND2 cells, which were exposed for 0.5-3h to TBMS1 or vehicle. We found that TBMS1 significantly reduced the expression of VEGFR2 in a dose-dependent manner (Figure 5a). This inhibitory effect could already be observed after a short exposure time of only 0.5h. In addition, we detected lower expression levels of Tie2 in eEND2 cells, which were treated for longer time spans of 2-3h with TBMS1 (Figure 5a). In line with these findings, the important downstream signaling pathway AKT/mTOR was significantly down-regulated by TBMS1 treatment (Figure 5a). Besides, we analyzed the effects of TBMS1 on the expression of two other endothelial receptor tyrosine kinases, i.e. VEGFR1 and Tie1. Our results showed that 3h after treatment with 10μM TBMS1, the expression of VEGFR1 was only slightly reduced in eEND2 cells and Tie1 expression was not affected at all (Figure 5b). In contrast, expression of VEGFR2 and Tie2 was markedly suppressed (Figure 5b), indicating that TBMS1 preferentially inhibits the expression of these two proteins.

**Figure 6:**
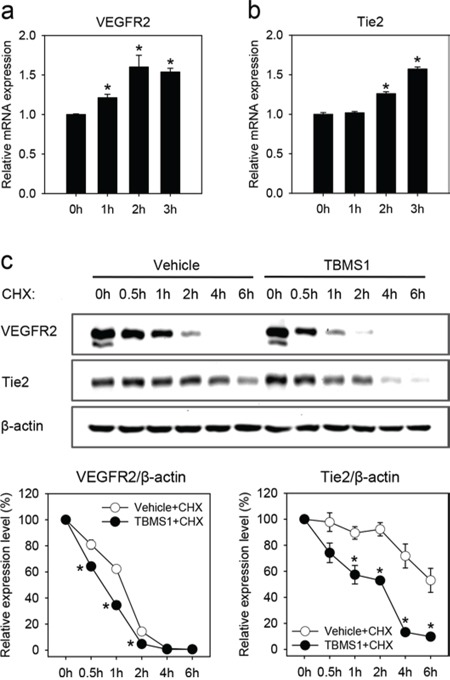
**a-b.** Relative VEGFR2 (a) and Tie2 (b) mRNA expression of eEND2 cells, which were treated with 10μM TBMS1 for 0, 1, 2 and 3h, as assessed by quantitative real-time PCR. The data were quantified from 3 independent experiments. Means ± SEM. *P<0.05 vs. 0h. **c.** Western blot analysis of VEGFR2 and Tie2 expression of eEND2 cells (% of time point 0h), which were treated with vehicle (white circles) or 10μM TBMS1 (black circles) in the presence of 100μM CHX for 0, 0.5, 1, 2, 4 and 6h. The data were quantified from 3 independent experiments. Means ± SEM. *P<0.05 vs. vehicle.

To clarify how TBMS1 reduces the cellular protein levels of VEGFR2 and Tie2, we performed quantitative real-time polymerase chain reaction (PCR) analyses of vehicle-treated and TBMS1-treated eEND2 cells. We found that exposure to TBMS1 increased the relative mRNA expression levels of VEGFR2 (Figure 6a) and Tie2 (Figure 6b) in these cells. This finding indicates that the compound has no inhibitory effects on the transcription and mRNA stability of VEGFR2 and Tie2.

We next analyzed the action of TBMS1 on VEGFR2 and Tie2 protein expression in the presence of cycloheximide (CHX), which suppresses the new synthesis of proteins. We could demonstrate that the degradation of already present VEGFR2 and Tie2 was markedly accelerated in TBMS1-treated eEND2 cells when compared to vehicle-treated controls (Figure 6c).

**Figure 7:**
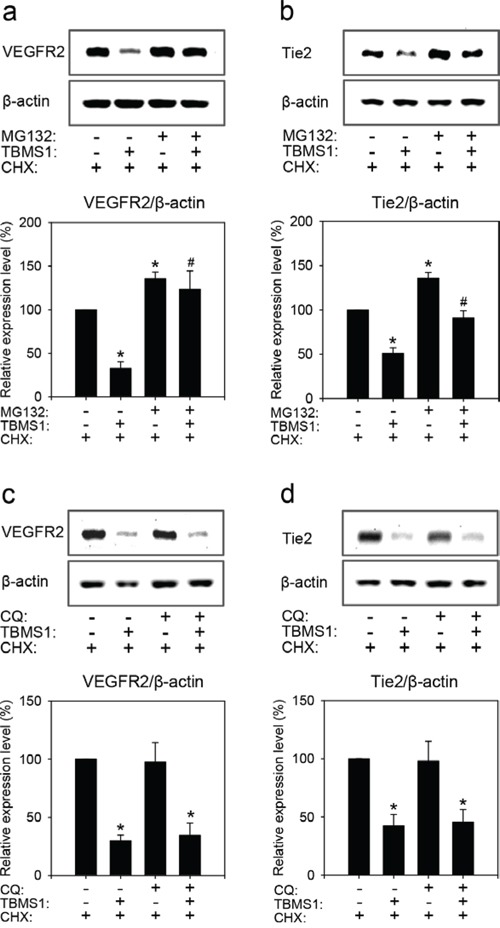
Western blot analysis of VEGFR2 **(a, c)** and Tie2 **(b, d)** expression of eEND2 cells (% of control). The cells were pretreated without or with 30μM MG132 or 100μM CQ for 2h, and then incubated with vehicle (distilled water) or 10μM TBMS1 in the presence of 100μM CHX for another 1h (a, c) or 4h (b, d). The data were quantified from 3 independent experiments. Means ± SEM. *P<0.05 vs. vehicle + CHX (control); ^#^P<0.05 vs. TBMS1 + CHX.

Proteasomes and lysosomes are the two major protein degradation systems in eukaryotic cells. To determine which one of them contributes to the observed TBMS1-induced degradation of VEGFR2 and Tie2, we pretreated eEND2 cells for 2h with the proteasome inhibitor (R)-MG-132 (MG132) or the lysosome inhibitor chloroquine diphosphate salt (CQ) before vehicle or TBMS1 treatment. We could show that MG132 significantly reversed TBMS1-induced degradation of VEGFR2 (Figure 7a) and Tie2 (Figure 7b). This, however, was not observed after CQ exposure (Figure 7c and 7d). These results indicate that TBMS1 promotes VEGFR2 and Tie2 degradation exclusively via the proteasome.

### TBMS1 action on VEGFR2 and Tie2 expression in tumor microvessels

**Figure 8:**
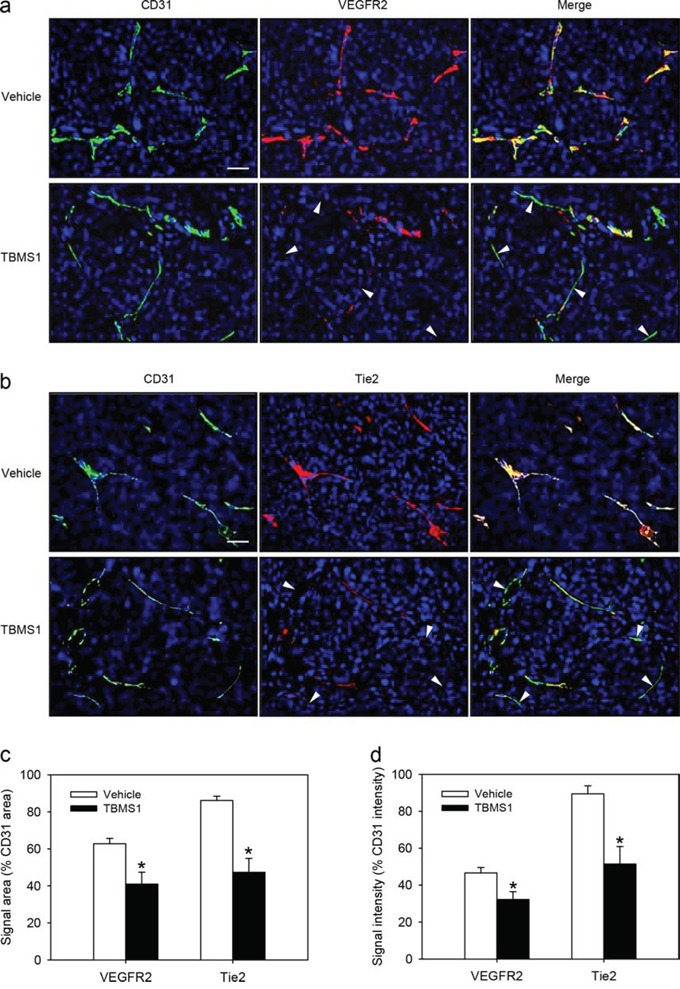
**a, b.** Immunohistochemical detection of VEGFR2 (a, red) and Tie2 (b, red) of CD31-positive microvessels (a, b, green) in a NCI-H460 flank tumor from a vehicle-treated control mouse and a TBMS1-treated animal. Sections were stained with Hoechst 33342 to identify cell nuclei (blue). Arrowheads indicate microvessels with reduced VEGFR2 or Tie2 expression. Scale bars: 35μm. **c, d.** Quantification of the signal area (% CD31 area) (c) and signal intensity (% CD31 intensity) (d) of microvascular VEGFR2 and Tie2 expression in vehicle-treated and TBMS1-treated tumors. The data were quantified from 8 mice per group. Means ± SEM. *P<0.05 vs. vehicle.

To confirm our mechanistic *in vitro* results in our *in vivo* NSCLC xenograft model, we finally performed double immunohistochemical stainings of CD31/VEGFR2 and CD31/Tie2 within the NCl-H460 flank tumors of vehicle-treated and TBMS1-treated mice. We could demonstrate that both VEGFR2 and Tie2 expression levels were markedly reduced in CD31-positive microvessels of TBMS1-treated tumors when compared to vehicle-treated controls (Figure 8a-8d).

## DISCUSSION

An increasing number of studies indicates that TBMS1 posseses anti-cancer activity [18–22]. So far, this has been attributed to anti-proliferative and pro-apoptotic effects of the compound on different cancer cell lines [1, 4, 23]. Moreover, TBMS1 causes mitochondrial dysfunction and endoplasmatic reticulum stress [24, 25]. In the present study, we demonstrate an important novel anti-tumor mechanism of TBMS1, i.e. inhibition of angiogenesis.

We first analyzed the anti-tumor action of TBMS1 in a NSCLC xenograft model. Treatment with the compound suppressed the growth of NCl-H460 tumors. Immunohistochemical analyses further revealed that TBMS1-treated tumors exhibit a lower microvessel density when compared to vehicle-treated controls. However, under these *in vivo* conditions it was difficult to clarify whether the latter observation was the main reason for the reduced tumor growth or the consequence of a decreased proliferation rate of the tumor cells. Therefore, we additionally analyzed the action of TBMS1 in a rat aortic ring assay. This *ex vivo* angiogenesis assay bears the major advantage that the anti-angiogenic action of a compound can be analyzed independently from the influence of surrounding tumor cells. Accordingly, the aortic ring assay is frequently used for the identification of novel anti-angiogenic agents [26–28]. By this assay we could demonstrate that TBMS1 dose-dependently suppresses the formation of vascular sprouts.

We next analyzed *in vitro* the effect of TBMS1 on the viability and cell death of eEND2 cells, HDMEC, NCl-H460 and A549 cells. We found that endothelial cells of both murine and human origin reacted much more sensitive to TBMS1 than the tumor cells. This indicates that inhibition of angiogenesis may represent the major mechanism of the anti-tumor activity of TBMS1. Our *in vitro* data further demonstrate that TBMS1 exerts pleiotropic anti-angiogenic effects in endothelial cells, which not only involve the induction of apoptosis, but also the inhibition of their migratory activity. Phalloidin staining of the cytoskeleton of eEND2 cells showed that the latter observation may be caused by the disturbance of actin filament organization, which is essentially involved in cellular migration [29].

We also investigated the molecular mechanisms underlying the anti-angiogenic effects of TBMS1. For this purpose, we focused on the two important angiogenesis signaling pathways, i.e. the VEGF/VEGFR2 and the Ang-2/Tie2 axis. We found that TBMS1 interferes with both systems. Western blot analyses revealed that short-term exposure to TBMS1 markedly reduces the endothelial expression of VEGFR2 and Tie2, which is associated with down-regulation of AKT/mTOR signaling. This mode of action offers the possibility to inhibit different steps of tumor angiogenesis. In fact, the vascularization of a tumor initially requires the destabilization of the surrounding normal vasculature, which is promoted by Ang-2/Tie2 signaling [30]. Subsequently, the endothelial cells of the microvessels are stimulated by VEGF/VEGFR2 signaling to proliferate and migrate into the tumor tissue [31]. Hence, the combined blockade of these processes may markedly contribute to the anti-angiogenic efficiency of TBMS1, comparable to the modern concept of Ang-2 and VEGF axes combination therapies in clinical oncology [14].

Intracellular protein expression levels are determined by transcription, mRNA stability, translation and degradation. We herein found that TBMS1 does not inhibit the transcription and mRNA stability of endothelial VEGFR2 and Tie2, but promotes the degradation of these proteins. Previous studies could demonstrate that similar to TBMS1, VEGF also causes rapid degradation of VEGFR2 [32–34]. The mechanism of VEGF-induced VEGFR2 degradation is controversially discussed. The majority of studies indicates that VEGF induces the lysosomal degradation of VEGFR2 [32, 33]. However, there are other reports suggesting that the degradation of VEGFR2 can also occur in the proteasome [34, 35]. In line with the latter reports, we found that VEGFR2 is degraded by the proteasome and that this degradation is enhanced by TBMS1.

So far, the degradation of Tie2 is poorly understood. Although it has been reported that Ang-1 rapidly stimulates Tie2 ubiquitination and degradation [36], the primary system for its degradation has not yet been identified. Based on our novel results we now suggest that it is the proteasome. In fact, we could demonstrate that treatment of endothelial cells with the proteasome inhibitor MG132 increased Tie2 expression. Moreover, MG132 reversed the inhibitory effect of TBMS1 on endothelial Tie2 expression.

Taken together, the present study demonstrates that TBMS1 inhibits angiogenesis, which is based on its stimulatory action on proteasomal VEGFR2 and Tie2 degradation. Accordingly, TBMS1 acts as a pleiotropic anti-angiogenic compound, which suppresses multiple mechanisms of the angiogenic process. Hence, TBMS1 represents a promising phytochemical for the establishment of novel anti-angiogenic treatment strategies against cancer and other angiogenesis-related diseases.

## MATERIALS AND METHODS

### Chemicals

TBMS1, CHX and MG132 were purchased from Santa Cruz Biotechnology (Heidelberg, Germany). CQ was purchased from Sigma-Aldrich (Taufkirchen, Germany).

### Cell culture

The murine endothelial cell line eEND2 (kind gift from the Department of Surgery, Malmö Hospital, Lund University, Malmö, Sweden) was cultured in Dulbecco's modified Eagle's medium (DMEM; PAA, Cölbe, Germany) supplemented with 10% fetal calf serum (FCS), 100U/mL penicillin and 0.1 mg/mL streptomycin (PAA). The human NSCLC cell lines NCI-H460 and A549 (ATCC, Wesel, Germany) were maintained in RPMI 1640 medium supplemented with 10% FCS, 100U/mL penicillin and 0.1 mg/mL streptomycin. HDMEC (PromoCell, Heidelberg, Germany) were cultured in endothelial cell growth medium-MV (EGM-MV; PromoCell). All cells were cultured at 37°C in a humidified atmosphere containing 5% CO_2_.

### Ethical statement

All animal experiments were performed in accordance with the German legislation on protection of animals, the EU Directive 2010/63/EU and the NIH Guidelines for the Care and Use of Laboratory Animals (NIH Publication #85-23 Rev. 1985) and were approved by the local governmental animal care committee.

### NSCLC xenograft model

The effects of TBMS1 on tumor growth and angiogenesis were analyzed in a NSCLC xenograft model. For this purpose, 16 female CD1 nu/nu mice (age: 6-8 weeks; body weight: 20-22g) were anesthetized by intraperitoneal (i.p.) injection of ketamine (75 mg/kg body weight; Ursotamin; Serumwerk Bernburg, Bernburg, Germany) and xylazine (15 mg/kg body weight; Rompun; Bayer, Leverkusen, Germany). Then, 1 × 10^6^ NCI-H460 cells were subcutaneously injected as a single cell suspension in phosphate buffered saline (PBS) into each flank of the animals. The mice were randomly divided into 2 groups, which were daily treated with an i.p. injection of vehicle (100μL 0.9% NaCl; control, n=8) or TBMS1 (5 mg/kg, n=8). Two perpendicular diameters of the developing tumors were repetitively measured at day 0 (baseline), 3, 7, 10, 14 and 17 (endpoint due to excessive tumor size in control animals) by means of a caliper. The tumor volumes were calculated using the formula V = 1/2 (L × W^2^), where L was the longest and W the shortest diameter [37]. At the end of the *in vivo* experiments, the mice were sacrificed with an overdose of the anesthetics and the tumors were carefully excised, weighed and further processed for immunohistochemical analyses.

### Immunohistochemistry

Formalin-fixed specimens of the tumors were embedded in paraffin. Subsequently, 2μm-thick sections were cut and stained with a rat monoclonal anti-CD31 antibody (1:30; Dianova GmbH, Hamburg, Germany), a rabbit polyclonal anti-VEGFR2 antibody (1:100; Cell Signaling Technology, Frankfurt am Main, Germany) or a goat polyclonal anti-Tie2 antibody (1:100; R&D Systems, Wiesbaden, Germany) followed by a goat-anti-rat IgG Alexa Fluor488-labeled (1:50; Life Technologies, Darmstadt, Germany), a Cy3-conjugated goat-anti-rabbit IgG secondary antibody (1:50; Dianova GmbH) or a Cy3-conjugated donkey-anti-goat IgG secondary antibody (1:50; Dianova GmbH). Cell nuclei were stained with Hoechst 33342 (1:500; Sigma-Aldrich). Sections were subsequently examined using a BX60 epifluorescence microscope (Olympus, Hamburg, Germany). For the quantitative analysis of the microvessel density (mm^−2^), numbers of CD31-positive microvessels were counted in four microscopic regions of interest (ROIs) in the periphery and in one ROI in the center of each tumor at 10x magnification. Microvascular VEGFR2 and Tie2 expression was measured in additional five randomnly chosen ROIs of each tumor by quantifying the signal area (% CD31 area) and signal intensity (% CD31 intensity) using Image J software (US National Institutes of Health, Bethesda, MD, USA).

### Aortic ring assay

To study the effects of TBMS1 on angiogenesis in an experimental setting excluding the influence of tumor cells, an aortic ring assay was performed as previously described in detail [38, 39]. Briefly, aortic rings from 2 female Sprague Dawley rats were embedded in Matrigel (Corning, Wiesbaden, Germany). After polymerization of the Matrigel, DMEM containing 0 (vehicle), 2.5, 5 and 10μM TBMS1 was added. The aortic rings were incubated at 37°C for 6 days with medium change on day 3, followed by observation with phase-contrast microscopy (BZ-8000; Keyence, Osaka, Japan). The area (mm^2^) and the maximal length (μm) of the outer aortic vessel sprouting were quantified using the software CapImage (version 8.5; Zeintl, Heidelberg, Germany). In this assay, 8 aortic rings per group were analyzed.

### WST-1 assay

To assess the effects of TBMS1 on the viability of eEND2 cells, HDMEC, NCI-H460 and A549 cells, WST-1 assays (Roche Diagnostics, Mannheim, Germany) were performed according to the manufacturer's instructions. Briefly, 5 × 10^3^ cells were seeded in 96-well plates and were treated with vehicle (distilled water, control) or serial dilutions of TBMS1. After 24h, 10μL WST-1 reagent per 100μL medium was added into each well. After 30min incubation at 37°C, the absorbance of each well was measured at 450 nm with 620 nm as reference using a microplate reader (PHOmo, anthos Mikrosysteme GmbH, Krefeld, Germany). A value of 100% was assigned to the control group, and the concentration of TBMS1 that reduced the number of viable cells to 50% of its maximal inhibitory effect (IC50) was derived by an interpolate logaritmic concentration curve. All WST-1 assays were performed with 4 repeats in 3 independent experiments.

### Flow cytometry

The effect of TBMS1 on necrotic and apoptotic cell death of eEND2 and NCI-H460 cells was analyzed by means of flow cytometry in 3 independent experiments. For this purpose, 3 × 10^5^ eEND2 or NCI-H460 cells were seeded in 6-well plates and treated with 0 (vehicle), 5 and 10μM TBMS1. After 24h, the cells were stained with annexin V and propidium iodide using an Annexin-V-FLUOS Staining Kit (Roche) according to the manufacturer's protocol. For this purpose, the cells were washed with cold PBS and resuspended in 400μL incubation buffer. The cells were then incubated with 5μL annexin V and 1μL propidium iodide (100μg/mL) for 15min in the dark at room temperature. Subsequently, flow cytometric analyses were performed using a FACScan Instrument (BD Biosciences, Heidelberg, Germany).

### Cell migration assays

The effects of TBMS1 on endothelial cell motility were tested by means of two different migration assays, i.e. the transwell migration assay and the scratch wound healing assay.

The transwell migration assay was performed as described previously [38] with minor modifications. Briefly, eEND2 cells maintained in the culture dish were treated with 0 (vehicle), 2.5, 5 and 10μM TBMS1 for 24h. Then, 2 × 10^5^ treated cells in 500μL FCS-free DMEM were seeded into the 24-well insert, and 750μL DMEM supplemented with 1% FCS was added to the lower well. Cells were subsequently allowed to migrate across a polyvinylpyrrolidone-coated polycarbonate filter with a pore size of 8μm (BD Biosciences) for 5h at 37°C. Migrated cells on the bottom side of the filter were fixed with methanol and stained with Dade Diff-Quick (Dade Diagnostika GmbH, Munich, Germany). The number of migrated cells was counted in 20 ROIs at 20x magnification (BZ-8000; Keyence) and expressed as percentage of the number of migrated cells in relation to vehicle-treated controls. For each TBMS1 concentration tested, the assay was performed in quadruplicate.

For the scratch wound healing assay, 3 × 10^5^ eEND2 cells in 500μL medium were seeded on a sterilized slide in a 100mm culture dish and allowed to attach for 5h, before 10mL DMEM medium was added. After 24h, the confluent cell monolayer was scraped with a white pipette tip (10μL) to generate 4 scratch wounds (i.e. 4 repeats per group) on each slide and rinsed twice with PBS to remove non-adherent cells. Then, fresh medium containing 0 (vehicle), 2.5, 5 and 10μM TBMS1 was added. Phase contrast microscopic images were taken immediately after scratching (0h), as well as after 12, 24 and 36h. Cell migration was determined as the wound closure rate, i.e. (original wound area - wound area at timepoint X)/original wound area ×100%.

### Phalloidin staining

To investigate the effects of TBMS1 on actin skeleton organization of eEND2 cells, we stained F-actin with phalloidin. For this purpose, eEND2 cells (1 × 10^4^ per well) seeded on glass coverslips in 24-well plates were exposed to vehicle or 10μM TBMS1. After 24h, the cells were fixed in 4% formalin for 10min at room temperature, washed twice in PBS and permeabilized for 10min with 0.2% Triton X-100, and then blocked in 1% bovine serum albumin (BSA) for 30min. Cells were then stained with Alexa Fluor 568-conjugated phalloidin (Invitrogen, Darmstadt, Germany) and Hoechst 33342 (Sigma-Aldrich) at room temperature for 30min and 10min, respectively. After two washes in PBS, slides were mounted in Kaiser's glycerol gelatin (Merck, Darmstadt, Germany) for fluorescence microscopy (BZ-8000; Keyence). The number of cells with stress fibers was assessed as a percentage of the total cell number. The analyses were performed with 3 repeats in 3 independent experiments.

### Western blot analysis

For Western blot analyses cells were lysed with RIPA buffer (Thermo Scientific, Bremen, Germany) containing 0.5mM phenylmethylsulfonyl fluoride (PMSF) and Protease Inhibitor Cocktail (1:75 v/v; Sigma-Aldrich) on ice for 5min. The lysate was then collected and centrifuged for 15min at 13,000 x g (4°C). The supernatant was saved as whole protein fraction. Protein concentrations were determined using the Pierce BCA Protein Assay Kit (Thermo Scientific) with BSA as standard. Then, 15μg protein/lane were separated on 8% sodium dodecyl sulfate (SDS) polyacrylamide gels and transferred to a polyvinylidene difluoride (PVDF) membrane (BioRad, Munich, Germany). After blockade of non-specific binding sites, membranes were incubated overnight at 4°C with a rabbit monoclonal anti-VEGFR2 antibody (1:500; Cell Signaling Technology), a goat polyclonal anti-Tie2 antibody (0.3μg/mL; R&D Systems), a rabbit polyclonal anti-phosphorylated (p)-AKT1/2/3 antibody (Thr308; 1:100; Santa Cruz Biotechnology, Heidelberg, Germany), a rabbit monoclonal anti-AKT antibody (1:500; Cell Signaling Technology), a rabbit monoclonal anti-p-mTOR antibody (Ser2448; 1:500; Cell Signaling Technology), a rabbit monoclonal anti-mTOR antibody (1:500; Cell Signaling Technology), a rabbit polyclonal anti-VEGFR1 antibody (1:100; Santa Cruz Biotechnology), a rabbit polyclonal anti-Tie1 antibody (1:100; Santa Cruz Biotechnology) or a mouse monoclonal anti-β-actin antibody (1:2,000, Sigma-Aldrich) followed by the corresponding horseradish peroxidase (HRP)-conjugated secondary antibodies (1:3,000; GE Healthcare, Freiburg, Germany). Protein expression was visualized with ECL Western blotting substrate (GE Healthcare) and images were acquired using a Chemocam device (Intas, Göttingen, Germany). The intensity of immunoreactivity was assessed using Image J software (US National Institutes of Health). All Western blot analyses were performed in 3 independent experiments.

### Quantitative real-time PCR

To test the effects of TBMS1 on the mRNA expression levels of VEGFR2 and Tie2, total RNA was extracted from eEND2 cells that were treated with 10μM TBMS1 for 0, 1, 2 or 3h by using RNeasy Mini kit (Qiagen, Hilden, Germany) following the manufacturer's instructions. In the reverse transcription reaction 1μg total RNA was processed using QuantiTect Reverse Transcription Kit (Qiagen). Quantitative real-time PCR was performed and analyzed in a MiniOpticon Real-Time PCR System (BioRad, München, Germany) with QuantiTect SYBR green PCR kit (Qiagen). The specific primer sequences were as follows: 5′-TTTGGCAAATACAACCCTTCAGA-3′ (forward) and 5′-GCTCCAGTATCATTTCCAACCA-3′ (reverse) for mouse VEGFR2; 5′-GAGTCAGCTTGCTCCTTTATGG-3′ (forward) and 5′-AGACACAAGAGGTAGGGAATTGA-3′ (reverse) for mouse Tie2; 5′-AGGTCGGTGTGAACGGATTTG-3′ (forward) and 5′-TGTAGACCATGTAGTTGAGGTCA-3′ (reverse) for mouse GAPDH. All PCR analyses were performed with 3 repeats in 3 independent experiments.

### Statistics

After testing the data for normal distribution and equal variance, differences between two groups were analyzed by the unpaired Student's t-test. Differences between multiple groups were analyzed by ANOVA followed by the Student Newman Keuls test with correction of the alpha error according to Bonferroni probabilities to compensate for multiple comparisons (SigmaStat; Jandel Corporation, San Rafael, CA, USA). All values are expressed as means ± SEM. Statistical significance was accepted for a value of P<0.05.
